# Biocrust Amendments to Topsoils Facilitate Biocrust Restoration in a Post-mining Arid Environment

**DOI:** 10.3389/fmicb.2022.882673

**Published:** 2022-07-26

**Authors:** Nick L. Schultz, Ian R. K. Sluiter, Geoffrey G. Allen, Nathali M. Machado-de-Lima, Miriam Muñoz-Rojas

**Affiliations:** ^1^The Future Regions Research Centre, Federation University Australia, Ballarat, VIC, Australia; ^2^School of Geography Earth and Atmospheric Sciences, The University of Melbourne, Parkville, VIC, Australia; ^3^Ogyris Ecological Research, Birdwoodton, VIC, Australia; ^4^School of Biological, Earth and Environmental Sciences, Centre for Ecosystem Science, University of New South Wales Sydney, Kensington, NSW, Australia; ^5^Department of Plant Biology and Ecology, University of Seville, Seville, Spain

**Keywords:** arid zone, mining rehabilitation, psyllium husk powder, soil cryptogamic biocrust, soil stabilization, cyanobacteria

## Abstract

Soil cryptogamic biocrusts provide many ecological functions in arid zone ecosystems, though their natural reestablishment in disturbed areas is slow. Accelerating reestablishment of biocrusts may facilitate the establishment of vascular plant communities within the timeframes of restoration targets (typically 5–15 years). One technique is to inoculate the soil surface using slurries of biocrust material harvested from another site. However, this is destructive to donor sites, and hence the potential to dilute slurries will govern the feasibility of this practice at large spatial scales. We conducted a replicated experiment on a disturbed mine site to test the individual and combined effects of two strategies for accelerating soil cryptogamic biocrust reestablishment: (1) slurry inoculation using biocrust material harvested from native vegetation; and (2) the use of psyllium husk powder as a source of mucilage to bind the soil surface, and to potentially provide a more cohesive substrate for biocrust development. The experiment comprised 90 experimental plots across six treatments, including different dilutions of the biocrust slurries and treatments with and without psyllium. Over 20 months, the reestablishing crust was dominated by cyanobacteria (including *Tolypothrix distorta* and *Oculatella atacamensis*), and these established more rapidly in the inoculated treatments than in the control treatments. The inoculated treatments also maintained this cover of cyanobacteria better through prolonged adverse conditions. The dilute biocrust slurry, at 1:100 of the biocrust in the remnant vegetation, performed as well as the 1:10 slurry, suggesting that strong dilution of biocrust slurry may improve the feasibility of using this technique at larger spatial scales. Psyllium husk powder did not improve biocrust development but helped to maintain a soil physical crust through hot, dry, and windy conditions, and so the potential longer-term advantages of psyllium need to be tested.

## Introduction

Soil cryptogamic biocrusts (hereafter biocrusts) are assemblages of microorganisms that form on the soil surface, including cyanobacteria, bacteria, mosses, liverworts, algae, lichen, and fungi (Eldridge and Greene, 1994). They are the dominant biological soil feature in arid and semi-arid landscapes (Metting, 1991). Biocrusts provide a suite of benefits to ecosystem function in arid landscapes. They stabilize the soil against wind and water erosion (Faist et al., 2017), influence rainfall infiltration (Chamizo et al., 2016), facilitate vascular plant establishment and survival (Havrilla et al., 2019), and can increase the nutrient status of soil, particularly through the fixation of nitrogen (N) by cyanobacteria (Delgado-Baquerizo et al., 2013; Machado de Lima et al., 2021).

Given their importance, the reestablishment of biocrusts after severe disturbances such as mining is essential to the restoration of self-sustaining and functional ecosystems. Natural reestablishment of biocrusts is a slow process and can take decades to millennia (Belnap and Eldridge, 2001) due to slow growth and limitations associated with propagules, aridity, low nutrient status and physical disturbance (Hawkes and Flechtner, 2002; Weber et al., 2016). It is now considered vital to facilitate this process to achieve restoration success in a timely manner (Zhao et al., 2016).

Biocrust reestablishment is commonly facilitated by inoculating the soil surface with biocrust material. There are several ways to achieve this (reviewed by Zhao et al., 2016), including the mass cultivation of biocrust propagules which are subsequently spread on the soil surface (Liu et al., 2013; Muñoz-Rojas et al., 2018). Another suite of techniques uses fragments of field-collected biocrust material that are incorporated into slurries for application to topsoil or that are implanted in the soil surface, and many studies have demonstrated the success of such techniques for increasing recovery rates (Belnap and Eldridge, 2001; Maestre et al., 2006; Bowker, 2007; Xiao et al., 2008; YanQin et al., 2009; Chiquoine, 2012), while others found that such techniques did not facilitate biocrust recovery (Chandler et al., 2019). Techniques that harvest biocrust from remnant ecosystems may have limited application for the broader global issue of biocrust reestablishment, as it requires one area to be compromised to benefit another. However, it is particularly suited to rehabilitation after mining, as the inoculation material can be collected from areas that are to be mined, and then returned to the mine path and disturbed ancillary areas after mining. As such, these inoculation techniques require further experimentation, particularly in arid zones and in Australia, where to our knowledge no field trials of facilitated biocrust inoculation have been performed. Furthermore, the findings of mesocosm studies need support from experiments under field conditions (such as Antoninka et al., 2020). Experiments also need to consider the feasibility of applying techniques at large spatial scales, and hence should test the effect of diluting slurries on biocrust establishment.

Unstable soil physical crust can limit biocrust reestablishment, and previous research has looked to increase the stability of soils to overcome this barrier to biocrust restoration. The use of polyacrylamide resins on soils has shown limited success (Davidson et al., 2002). Sand-binding shrub species can stabilize sandy soils and allow biocrust establishment (Li et al., 2004), and jute cloth can provide a substrate for lichen establishment (Condon and Pyke, 2016; Bowker et al., 2020; Slate et al., 2020), but such methods are expensive to implement at large spatial scales. The potential of psyllium as a soil stabilizer has also been recognized (Fick et al., 2020). Psyllium is derived from the seed husks of the perennial herb *Plantago ovata* and is a rich source of exopolysaccharide mucilage, which is a naturally occurring component of soils that plays an important role in stabilizing soil micro-aggregates. It is a component of several commercial soil stabilizing patents (Hubbs and Hubbs, 1998). However, psyllium has not been sufficiently tested in relation to biocrust reestablishment, nor in a range of environments and field conditions. In growth chambers, Blankenship et al. (2020) demonstrate that psyllium has considerable potential over other soil tackifiers like guar.

A strong driver of the focus on rehabilitating biocrust is the generally low success rate of dryland restoration (Ravi et al., 2010; Cortina et al., 2011). Restoration efforts have tended to focus primarily on putting vascular plants back into ecosystems (Sheoran et al., 2010; Vallejo et al., 2012), overlooking the role that biocrusts play in soil function and structure, and the hydrological function of soils (Duncan et al., 2020). The current study aims to test the use of biocrust slurry inoculation and the use of psyllium for improving the reestablishment of biocrusts after mining in an arid landscape in southwest New South Wales, Australia. We test the hypotheses that biocrust inoculation will, over the first 20 months, increase the cover of biocrust on the soil surface compared to control treatments, and that psyllium application will increase the cover of biocrust through increased stability of the soil physical crust. We also test if the concentration of biocrust material in the slurry influences biocrust reestablishment, as the dilution factor of slurry has a strong influence on the scale at which this technique could be sustainably employed. Finally, we assess the presence and composition of cyanobacteria in the reestablishing biocrust, as these organisms are characteristic of initial biocrust formation (Bowker, 2007).

## Materials and Methods

### Study Site and Experimental Design

**FIGURE 1:**
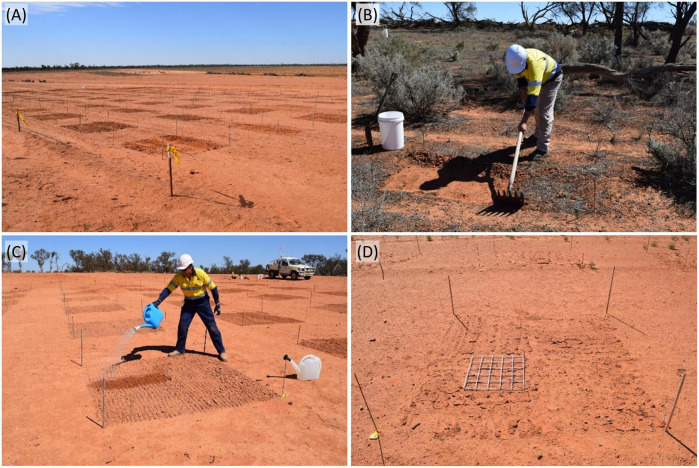
Biocrust trial on a former topsoil storage area: **(a)** ninety plots of 4 m^2^ were established with a buffer of 2 m separating each plot, **(b)** square plots (1 m^2^) of cryptogamic crust were harvested from nearby native vegetation to 1 cm depth with a rake-hoe, **(c)** each plot was raked lightly with a rake-hoe to provide micro-topography and hand-watered with 18 L of reverse osmosis water from a watering can, and **(d)** plot showing 0.25 m^2^ quadrat on the surface ready for monitoring using the center bamboo peg as a center pivot point; four of these 0.25 m^2^ quadrats were sampled per plot at each monitoring period.

The experimental site was a former topsoil stockpile area of the Gingko Mine (Figure 1), a mineral sands mine operated by Tronox Holdings PLC, approximately 35 km west of Pooncarie, in southwest New South Wales, Australia (33°22′S, 142°13′E). The study area has a hot desert climate according to the Koppen Classification System. Mean annual rainfall is 250 mm (BOM, 2018), although annual rainfall often falls below 200 mm for consecutive years (Environdata, 2019). Mean annual temperature is 18°C, although temperatures can range from 48 to –3°C (BOM, 2018). Evaporation rates (mean 5.6 mm.hr^–1^) are higher than rainfall across all months (Environdata, 2019). Soils of the study area are Pleistocene age aeolian deposits of the Woorinen Formation (Lawrence, 1966). Prior to mining, soils were predominantly Calcarosols (Isbell and NCST, 2016) with surface soil textures of plains dominated by sandy clay loam and clay loam, with subordinate dune areas often characterized by light sandy clay loam and sandy loam. Subsoil B-horizons are ubiquitously characterized by carbonate pedoderms with elevated calcium carbonate content.

The area was formerly cleared of remnant vegetation, with topsoil capped over the cleared land to a height of approximately 4 m. The stockpiled topsoil was then reclaimed for rehabilitation use. The local topsoil was retained across the plot area, but was not ripped and seeded as is standard mine practice for rehabilitating stockpile areas. The site had a near-smooth surface with a slope of 1–2° with a northeast-facing aspect. Ninety plots of 4 m^2^ each were established in a 10 × 9 grid, with plots 2 m apart (Figure 1). Each plot was marked using a metal pin with a numbered cattle tag in the western corner. The other three corners of each plot were pegged with bamboo stakes, and a short bamboo stake was placed in the center of each plot. The experimental site was protected from mine traffic with caution tape.

The experiment involved six treatments that comprised combinations of two amendment techniques: the application of biocrust slurry, and the application of psyllium husk powder as a soil stabilizer. Two different application rates of the slurry were used that represented one tenth and one hundredth of the biocrust found in the remnant ecosystem (these are referred to hereafter as the 1:10 and 1:100 biocrust amendments, respectively); the 1:10 amendment was comparable to rates applied in previous studies of slurry application (Fick et al., 2019, 2020), while the 1:100 amendment, to our knowledge, is a novel treatment designed to test if a diluted slurry can achieve similar results to the more concentrated treatment. Each of the 90 plots were randomly assigned one of the six treatments (15 replicates of each treatment), which were as follows: (1) control (no additions of biocrust or psyllium), (2) psyllium, (3) 1:10 biocrust amendment, (4) 1:10 biocrust + psyllium, (5) 1:100 biocrust amendment, and (6) 1:100 biocrust + psyllium.

In mid-January 2015, biocrust material for the amendment treatments was harvested from an untreated remnant belah (*Casuarina pauper*) woodland within 50 m of the experimental site. This woodland had high biocrust cover comprising moss, lichen and presumed algae and cyanobacteria. To calculate the amount of biocrust material to achieve 1:10 and 1:100 amendments, biocrust was collected by scraping 4 m^2^ to a depth of 0.5–1.0 cm with a rake hoe (Figure 1B) and shoveling the material into clean 20 L plastic buckets. The buckets of biocrust were stored in the shade for three days, after which all collected biocrust was bulked, mixed, and sieved through a 5-mm sieve to remove green vascular plant material, vascular plant fruits and cones, plant litter, and animal scats of red kangaroo (*Macropus rufus*), western gray kangaroo (*Macropus fuliginosus*) and feral goat (*Capra hircus*), and to break up larger pieces of biocrust. This produced 39 kg of biocrust from the 4 m^2^ area, allowing the amount of biocrust needed for each dilution treatment to be calculated. Additional biocrust material was collected in the same manner to provide the ∼130 kg of biocrust material needed for the experiment. The biocrust was stored for a further 2–3 days in the shade before weighing out samples for the experiment.

For each treatment, a clean, sealable plastic bag was prepared with the required biocrust and psyllium material. This included 3.9 kg (± 0.5 g) of biocrust for the 1:10 biocrust amendment treatments, 0.39 kg (± 0.5 g) for the 1:100 treatments, and 25 g of commercially available psyllium powder for each treatment that included psyllium. Biocrust amendments and psyllium were thoroughly mixed where applicable. The 25 g of psyllium represented an application rate of 62.5 kg.ha^–1^.

In early February 2015 (19 and 20 days after the biocrust was collected), the experimental plots were inoculated over two consecutive low wind days. All 90 4-m^2^ plots were raked to 10–20 mm depth to create a micro-topography for receiving the prepared amendments (Figure 1C). Amendments were dry spread by hand evenly over the 4 m^2^ plots and all plots were watered with 18 L of reverse osmosis water *via* a standard garden plastic watering can. This was the equivalent of a 4.5 mm rainfall event.

### Assessment of Biocrust Cover and Cyanobacteria Composition

All plots were monitored six, thirteen and twenty months after the initial treatments. A point quadrat method was used to monitor 1 m^2^ at the center of each plot; 25 permanent points in each plot were monitored using a grid. Each point in the grid was assessed for the presence of biocrust and physical soil crust. Biocrust was assessed based on visual inspection, with the color and texture of the soil surface indicating if biocrust species were present at each grid point. The physical soil crust was assessed using a firmness test; soil surface that could withstand being readily depressed with the index finger was deemed to have a physical soil crust (Burkett et al., 2011), with this force estimated to represent approximately 200 kPa (Collingham and Kan, 2019). For each plot, the total cover of any vascular vegetation that had established during the experiment was visually estimated (to the nearest 1%, where cover was < 5%, otherwise to the nearest 5%). All 90 experimental plots were photographed following inoculation and at each monitoring point at 6, 13, and 20 months. Rainfall, temperature, and wind speeds were logged continuously throughout the experiment by the weather station at Ginkgo Mine.

Samples of re-establishing crust were taken from three plots at the conclusion of the experiment, collecting the upper 15 mm of the soil surface using a metal spatula and collected in plastic containers. These were posted to the laboratories at the Center for Ecosystem Science (UNSW Sydney) to identify the main taxa of cyanobacteria comprising the crust. Samples were stored at 4°C for 1 week before processing.

Biocrusts samples were then rehydrated with distilled water and the visible biomass portions were inoculated in BG11 solid growth medium (Rippka et al., 1979) under light:dark cycles (16:8 at 28/20°C). In the “slurry + psyllium” treatments, we observed a wide coverage of filamentous cyanobacteria, which were not present in those samples inoculated with psyllium only (Supplementary Figure 1). Because of the poor colonization and lack of filamentous cyanobacteria in the psyllium-only cultures we couldn’t perform morphological nor molecular analyses in the “psyllium” treatment samples. The produced fascicles and mats of filamentous cyanobacteria from the “slurry + psyllium” treatments were analyzed through molecular analyses. DNA was extracted using the DNeasy PowerSoil Kit (Qiagen, Venlo, Netherlands) according to the manufacturer’s instruction. The 16S rRNA partial gene was amplified by polymerase chain reaction (PCR) using primers specific for cyanobacteria, CYA 359 and 781Ra/781Rb (Nübel et al., 1997), with 25 μL of total reaction volume, containing 12.5 μL 2X KAPA Taq Extra HotStart ReadyMix with dye, 1.25 μL of 359F primer (10 ρmol), 0.65 μL of each 781R primers (10 ρmol), 9.0 μL “Milli-Q” water, and 10 n g genomic DNA. Thermal cycling initial denaturation of 94°C for 5 min followed by 30 cycles of denaturation at 94°C for 1 min, annealing 55.5°C and extension at 72°C for 1 min and a final extension at 72°C for 7 min. The PCR products were analyzed on agarose gel with “GelRed 0.6X” (Biotium), viewed on transilluminator. Unpurified subsamples were submitted to the Ramaciotti Center for Genomics (UNSW, Australia) for PCR clean up, sequencing reaction and capillary separation. Sanger sequencing was performed using “BigDye Terminator (BDT) version 3.1” kit following the manufacturer’s protocol. Primers 359F, 781Ra, and 781Rb (Nübel et al., 1997) were used for sequencing. Consensus sequences were assembled into contigs using the “Phred/Phrap/Consed” software, and Phred over 20 were used (Ewing et al., 1998; Gordon et al., 1998; Erwin and Thacker, 2008). The sequences’ identification was determined by similarity using BLAST^1^.

### Data Analysis

Point quadrat data from each plot was used to calculate the cover of biocrust and physical soil crust in each plot. To test for differences in biocrust, physical soil crust and vegetation cover among treatments at each monitoring point non-parametric tests were used as the data failed the Shapiro-Wilk normality test. Kruskal-Wallis tests and Dunn tests were used for pairwise comparisons with a Benjamini-Hochberg *p*-value adjustment. Box-and-whisker plots were constructed to visualize the distribution of cover values for each treatment in each season. All data analysis and the construction of boxplots were performed using R (R Core Team, 2021).

## Results

### Weather Observations During the Experiment

**FIGURE 2:**
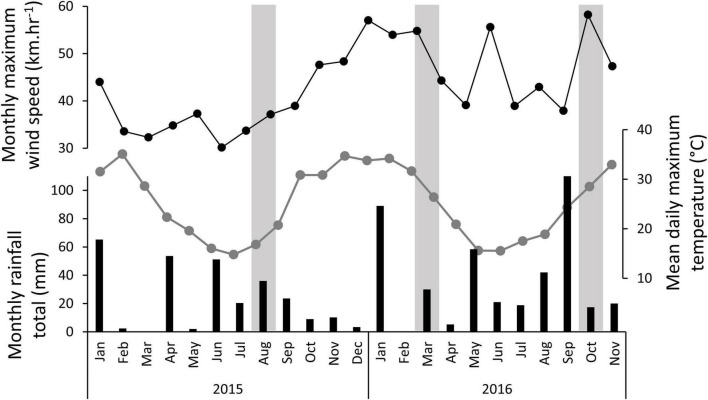
Climate variables throughout experiment showing monthly rainfall totals (black bars), the mean daily maximum air temperature for each month (connected grey dots), and the maximum wind speed recorded in each month (connected black dots). The vertical grey bars signify the months in which the experiment was monitored.

The monthly temperature, rainfall, and maximum wind speeds throughout the experiment are shown in Figure 2. The data shows substantial rainfall events in the months leading up to each of the three monitoring seasons. Wind speeds were persistently high in the 3 months leading up to the second monitoring point at 13 months, with a maximum wind speed of 57.0 km.hr^–1^, and average daily maximum wind speeds of 30.0–30.4 km.hr^–1^ for those months. Wind speeds were also high in the month of the final monitoring, with a maximum wind speed of 58.2 km.hr^–1^.

### Biocrust Cover

**FIGURE 3:**
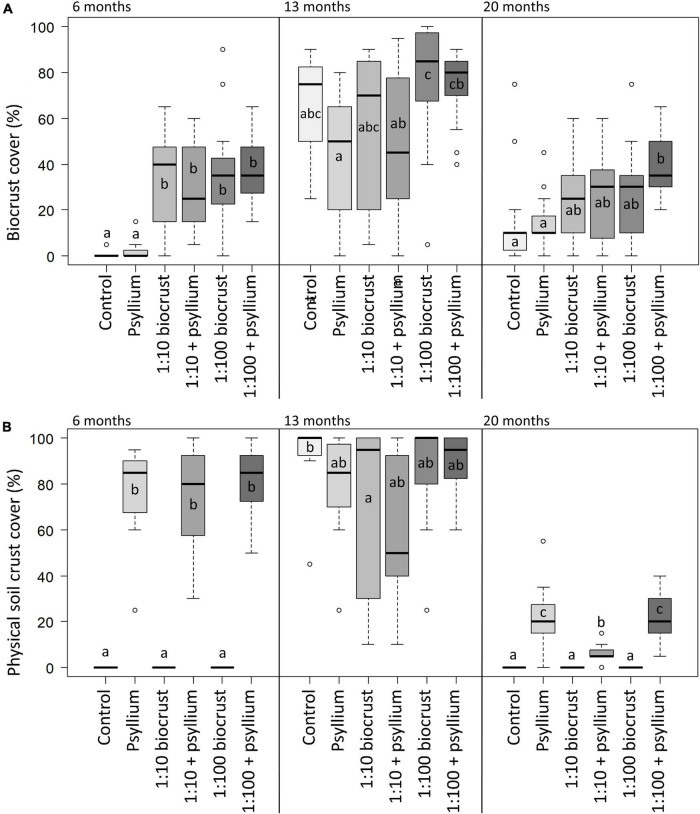
**(A)** Biocrust cover and **(B)** physical soil crust cover. Box and whisker plots representing the range of values for biocrust cover observed in each treatment, in each season. Boxes show the median (horizontal line) and interquartile range (top and bottom of the box). Whiskers show the furthest point that is within 1.5 times the interquartile range. Outliers are represented by hollow circles. Pair-wise comparisons of means were tested using Kruskal-Wallis and Dunn’s tests. Letters compare means among treatments within each monitoring season; treatments that do not share a common letter differ significantly (*P* < 0.05).

Across the three monitoring periods, all establishing biocrust was dominated by cyanobacteria. We found no visual evidence of the presence of later-successional crust taxa such as lichens or moss. There were significant differences in the establishment of biocrust among treatments and seasons (Figure 3). At the first monitoring point, after 6 months, the control and psyllium treatments showed a very low cover of biocrust (mean cover 0.7 ± 0.4% and 2.1 ± 0.4%, respectively), while the four treatments that included slurry inoculation had developed a substantial biocrust cover (mean cover 32 ± 6%, 29 ± 5%, 36 ± 6%, and 38 ± 4% for the “1:10 biocrust,” “1:10 biocrust + psyllium,” “1:100 biocrust” and “1:100 biocrust + psyllium” treatments, respectively). There were no significant differences in the mean biocrust cover among the four inoculation treatments, and all four had significantly greater biocrust cover than the control and psyllium treatments (*p* < 0.001 for each). At the second monitoring period, all six treatments demonstrated a high biocrust cover, although the “psyllium” treatment had significantly lower mean biocrust cover than the “1:100 biocrust” treatment (*z*^2^ = 3.4, *p* = 0.009) and “1:100 biocrust + psyllium” treatments (*z*^2^ = 2.9, *p* = 0.032; Figure 3A). The cover of biocrust decreased in all treatments between 13 and 20 months after establishment. However, this decrease was greater in the control and psyllium treatments than in the four inoculation treatments. The “1:100 crust + psyllium” treatment showed the highest mean biocrust cover after 20 months and had a significantly higher mean biocrust cover than the “control” treatment (*z*^2^ = 4.2, *p* < 0.001) and the “psyllium” treatment (*z*^2^ = 3.4, *p* = 0.004; Figure 3A).

**FIGURE 4:**
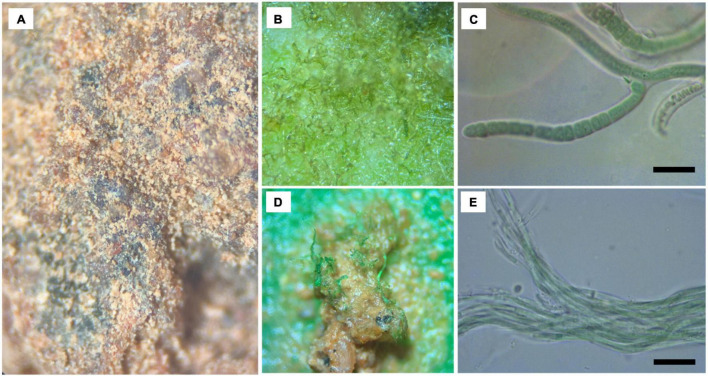
**(a)** Microscope view of regenerating biocrust and identified (most abundant) cyanobacteria from the regenerating biocrust sample. **(b,c)** Scytonemataceae filaments from culture conditions, and **(d,e)** Oscillatoriales filamentous from culture conditions. Scale bars in **(c,e)** are 20 μm.

Morphological analysis of a subset of biocrust samples from the “1:10 biocrust + psyllium” and “1:100 biocrust + psyllium” treatments showed that the establishing biocrusts were dominated by two cyanobacterial groups: (1) heterocystous cyanobacteria, with a population represented by false branched filaments with heterocytes, and (2) non-heterocystous cyanobacteria, with a population represented by homocystous unbranched filaments, densely arranged in parallel or forming spirally coiled fascicles, isodiametric to longer than wide cells and conical apical end cells (Figure 4). These two cyanobacterial groups were molecularly identified by triplicate, based on a 420 bp sequence generated by specific cyanobacteria primers (359F–781R). After a comparison with sequences in the NCBI database using the BLAST tool, the first strain showed 97.6 percentage identity with *Tolypothrix distorta* ATE717 (MK247975), while the second strain showed 99.0 percentage identity with *Oculatella atacamensis* ATE710 (MK248008).

### Physical Soil Crust Cover

There were significant differences in the physical soil crust cover among treatments and across the three monitoring points (Figure 4B). At the second monitoring point (13 months), all treatments had a coherent physical soil crust, with no significant differences among treatments. However, at the first and third monitoring points, only those treatments that included psyllium showed any cover of coherent physical soil crust. At the end of the trial, the three treatments that included psyllium maintained some cohesion in the physical soil crust, while the control treatment and the two inoculation treatments without psyllium had no cover of physical soil crust. This is despite the “1:10 biocrust” and “1:100 biocrust” treatments exhibiting a moderate cover of biocrust at the final monitoring point. It is important to note here that the biocrust observed in these treatments was comprised of cyanobacteria sitting on a non-cohesive soil surface. The dynamic nature of the physical soil crust is presumably related to climatic conditions, and this is discussed further below.

### Vascular Plant Cover

The mean cover of vascular plants across all plots at the final monitoring point was 17.3 ± 1.4%, and this cover was almost entirely provided by Ward’s weed (*Carrichtera annua*, Brassicaceae), an annual, exotic weed. There were no significant differences in vascular plant cover among the treatments at the final monitoring point (*z*^2^ = 10.0, *p* = 0.07), suggesting that none of the treatments favored vascular plant establishment over the time scale of the experiment. When the data were pooled across treatment types, there was no significant correlation between vascular plant cover and either biocrust (*R*^2^ = 0.001; *p* = 0.49) or physical soil crust (*R*^2^ = 0.013; *p* = 0.29). As such, there was no evidence that vascular plants influenced biocrust or physical soil crust, or vice versa, over the time scale of the experiment.

## Discussion

The results showed that plots inoculated with biocrust slurry established early-successional biocrust (cyanobacteria) more rapidly than control plots, and they maintained this crust better through adverse conditions. The results also show that a 10-fold dilution of the slurry had no impact on its effectiveness. Psyllium helped maintain a physical soil crust through hot and dry conditions. Each of these findings is discussed below.

### Slurry Inoculation

Six months after the establishment of the plots, only the inoculated treatments demonstrated a biocrust cover. This suggests the biocrust material in the slurry supplied the propagules necessary to colonize the soil surface. Maestre et al. (2006) found a similar result and used DNA analysis to confirm that cyanobacteria colonizing the soil surface after slurry inoculation were the same species present in the crust used for the slurry. We cannot confirm that the cyanobacteria we detected were in the slurry. We suspect it is the case, though an alternative explanation for our result is that the slurry provided nutrients to allow the growth of cyanobacteria that may have been present in the soil. Regardless, the results suggest slurry application has significant potential for accelerating biocrust development, though further considerations are necessary, as we will discuss.

A surprising result from the experiment was the high cover of biocrust across all treatments at the second monitoring point, 13 months after treatment application. Presumably, favorable conditions, including a large rain event in January 2016, allowed the proliferation of cyanobacteria over a short timeframe, and unlike the first monitoring period, this did not rely on the original supply of propagules in the slurry as the total cover was high in the two treatments without inoculation. However, by the third monitoring period the biocrust cover had decreased substantially across all treatments, but less so in the inoculated treatments—it seems the cover of cyanobacteria was more resilient and persistent in the treatments that had received inoculation. It is possible at the second monitoring period in the treatments that had not been inoculated, the cyanobacteria observed were different to those in inoculated treatments, and that these species were less robust to adverse conditions. Differing detectability of biocrust between seasons may have also played a part in the biocrust cover results. Temporal variability in the observed cyanobacteria cover can be influenced by vertical migration by cyanobacteria in the top 2 cm of the soil profile (Garcia-Pichel and Pringault, 2001) in response to changes in available moisture. At the second monitoring point, after significant rain events, the detectability of the biocrust may have been at its highest, amplifying the differences in biocrust cover detected between the seasons. In hindsight, it might have been useful to monitor plots before and after watering all plots, which may have accounted for the effects of soil moisture at the time of sampling.

Despite the decrease in biocrust cover between the second and third monitoring points, a pertinent aspect of the result is that the inoculated treatments maintained higher biocrust cover than the “control” and “psyllium” treatments. This result again attests to the potential of slurry inoculation in field conditions in arid environments. However, it is not clear if the biocrusts observed at the final monitoring point were comprised of the same species observed at monitoring point 1 and 2 or if other propagules in the slurry had subsequently colonized. The later is a possibility; Lan et al. (2021) describe how even within the cyanobacteria there is a successional trend from carbon-fixing cyanobacteria to nitrogen-fixing cyanobacteria. The two taxa identified at our final monitoring point (*Tolypothrix distorta* and *Oculatella atacamensis*) are nitrogen-fixing taxa that Lan et al. (2021) found to be more abundant in mid-successional biocrusts than in early-successional biocrusts.

It seems that the main short-term role of the biocrust inoculant is to provide propagules for the recolonization of cyanobacteria, as there was no evidence of the reestablishment of lichen or moss species. As such, laboratory-cultured cyanobacteria inoculum might more-efficiently provide this. Muñoz-Rojas et al. (2018) found that mine-site soils were rapidly colonized by mixtures of cyanobacteria cultures, improving soil organic carbon. These cyanobacteria inoculants can also biomineralize arid soils and promote water harvesting which can be beneficial to the neighboring microbiota (Jiménez-González et al., 2022). An additional consideration here is the potential role of microbes in the growth of cyanobacteria. Nelson and Garcia-Pichel (2021) demonstrate that cyanobacterial growth and cover after inoculation are improved when N-fixing diazotrophs are included in inoculum. We interpret that the absence of lichen and moss is due primarily to the temporal scale of the study—such species typically take > 5 years to re-establish (Belnap, 2006). However, the reason for their slow establishment is partly due to slow growth, but also due to a lack of suitable substrate on which to grow, and the early-successional cyanobacteria are generally thought to make the soil surface more amenable to lichen and moss (Belnap and Eldridge, 2001). Slate et al. (2020) observed lichen growth within 4 years of slurry application using a very similar method of biocrust harvest and slurry application. However, they used cloth on the soil surface as substrate for lichen.

The performance of the dilute slurry was an important and encouraging result. In no instances did the dilute 1:100 slurry perform worse than the 1:10 slurry. At the final monitoring point, the “1:100 crust + psyllium” had a higher mean biocrust cover, and a significantly higher physical soil crust cover than the “1:10 crust + psyllium” treatment. The result suggests that the presence of propagules might be more important than the number of propagules for rapidly initiating the growth of cyanobacteria after inoculation. The biocrust inoculation treatments of Fick et al. (2019, 2020) used a comparable amount of biocrust (0.75 and 1.5 kg.m^2^) to our 1:10 treatment (0.98 kg.m^2^), but to our knowledge this is the first study to trial a strongly diluted inoculation (one-hundredth of the biocrust in the remnant system, or 0.098 kg.m^2^). Assuming that our results are transferable to broad-scale application of slurry inoculation, the result significantly increases the feasibility of the slurry inoculation method. Our results also suggest further diluted slurry inoculations could be trialed to determine the point at which the benefits of inoculation begin to wane.

The cyanobacteria species observed regenerating in this study, and that we presume comprised most of the regenerating crust, have been shown to confer benefits to the regeneration of arid-zone vegetation. Román et al. (2018) demonstrate that soil covered by *Tolypothrix distorta* will have lower albedo, higher chlorophyll a content, and greater total organic carbon and total nitrogen. Moreover, the structured sheath layer around *Tolypothrix* trichomes has a positive effect in soil stabilization (Kvíderová et al., 2019). *Oculatella*, a narrow filamentous cyanobacteria, is considered essential for initial biocrust formation (Roncero-Ramos et al., 2019), and can improve soil structure and function and secrete filaments and polymers that help bind soil. *Oculatella* is regularly recorded in biocrusts in Europe and South America (Roncero-Ramos et al., 2019; Machado de Lima et al., 2021), but to date has few records in Australia. However, it is morphologically similar to *Leptolyngbya*, making it difficult to distinguish the two taxa on morphology alone, and it has only recently been described after molecular and cytomorphological analyses proved its separation from *Leptolyngbya sensu stricto* (Zammit et al., 2012). As such, it is likely to be abundant in arid Australian biocrusts despite the lack of previous records. It is important to note its presence due to its potential use in inoculation cultures (Antoninka et al., 2016). These findings provide assurance that the regenerating cyanobacteria in our study area will be beneficial to restoration efforts.

### Psyllium

The psyllium husk powder treatments helped maintain a physical soil crust cover through hot, dry, and windy conditions, such that at the final monitoring point only those treatments that included psyllium maintained a physical soil crust. This could suggest that the inoculation + psyllium treatments will be more resilient to future conditions. It is assumed that the physical soil crust will provide a buffer against erosion. However, the presence of psyllium did not result in a higher cover of biocrust at the final monitoring point. This was a similar result to that of Fick et al. (2020) who found that psyllium amendments did not influence biocrust development but did improve soil stability. As such, the longer-term effects of psyllium on biocrust require testing. The influence of psyllium on vascular plant establishment and survival are not evident from this study, and require further investigation if psyllium is to be incorporated into broad-scale rehabilitation plans. Furthermore, the role of psyllium might need to be considered in the context of the spatial arrangement of arid ecosystems, which generally include run-off zones (where water runs over the surface) and run-on zones, or islands of fertility (Ludwig et al., 2005). Fick et al. (2019) found that plots with psyllium amendments had greater infiltration and lower run-off than both plots with biocrust amendment only and control plots (no treatments). Such factors should be considered if the aim is to restore heterogeneous environments that resemble the remnant arid vegetation. Despite the knowledge gaps about psyllium that remain, there appears to be significant potential for it to play a role in future restoration efforts.

Only one application rate of psyllium was used in this experiment (62.5 kg.ha^–1^). Blankenship et al. (2020) trialed three psyllium application rates (50, 100, and 200 kg.ha^–1^) in a mesocosm experiment. They found that psyllium increased the growth of the dryland moss *Bryum* sp., and that this result was somewhat proportional to the concentration of psyllium applied. Fick et al. (2020) applied 600 kg.ha^–1^ of psyllium. Further trials are needed to determine application rates for psyllium that will optimize its benefit to restoration efforts.

### Vascular Plant Cover

There was no evidence that any treatments influenced vascular plant cover during the experiment. Havrilla and Barger (2018) demonstrate in a hot arid ecosystem that biocrust has a negative influence on exotic herbaceous species, while having neutral or mixed effects on native herbaceous species. Other studies have similarly shown that biocrust might confer a resistance to exotic weed invasion (Condon and Pyke, 2018; Taylor, 2021). However, these findings seem context-dependent, and the influence of different components of biocrust (cyanobacteria, lichen, liverwort, and mosses) on different vascular plant taxa is unresolved. In our experiment, we suspect both the spatial, and temporal scales were not sufficient for a rigorous test of the effect of treatments on plant cover, particularly given the sparse nature of plant recruitment, the size of native shrubs relative to the plot sizes, and the slow recruitment of vegetation, particularly in the absence of seed addition. A more viable experiment to test the influence of biocrust on plant recruitment in this system might include larger plots to accommodate the spatial scale at which plant recruitment and the organization of resources occurs in arid zones. Alternatively, highly controlled experiments observing the interaction between seeds and different biocrust species might be revealing.

### Other Considerations

Other methods of accelerating biocrust establishment have been trialed in previous experiments. Maestre et al. (2006) implanted fragments of crust into plots, though this method did not prove as effective as the use of slurries. Fick et al. (2020) also found limited success of crust implants; the implanted plots had greater biocrust cover than control plots, but the cover was less than the cover that was implanted. Given that crust implants are more labor-intensive than slurry application, we suspect this is not a viable option for use in arid southwest NSW.

A factor that was not tested in this experiment is the influence of nutrient status on biocrust reestablishment. Bowker et al. (2005) showed, on the Colorado Plateau (United States), that micronutrients such as manganese and zinc can limit biocrust reestablishment. It would be useful to test if this is a limiting factor to biocrust growth and reestablishment in Australia’s arid zones, particularly as zinc is present in very low amounts (average 0.28 mg/kg; range 0.1–0.6 mg/kg; *n* = 20 samples) in the calcarosol soils at the Ginkgo Mine and across the study region generally.

## Conclusion

The study has provided data that suggest that both slurry and psyllium amendments could play a role in arid zone biocrust reestablishment. The performance of the dilute slurry lends support to the idea that slurry amendments might viably be applied at larger scales without compromising existing areas with well-developed biocrust. Psyllium showed considerable potential to improve the coherence of the physical soil crust, but the longer-term implications of psyllium amendments require further investigation.

## Data Availability Statement

The datasets presented in this study can be found in online repositories. The names of the repository/repositories and accession number(s) can be found in the article/Supplementary Material.

## Author Contributions

IS conceived the research. IS, GA, and NS designed the research. IS and GA conducted the field experiment and monitoring protocol. MM-R and NM-d-L conducted laboratory work. NS analyzed the data. IS, GA, NS, MM-R, and NM-d-L wrote and edited the manuscript. All authors contributed to the article and approved the submitted version.

## Conflict of Interest

The authors declare that the research was conducted in the absence of any commercial or financial relationships that could be construed as a potential conflict of interest.

## Publisher’s Note

All claims expressed in this article are solely those of the authors and do not necessarily represent those of their affiliated organizations, or those of the publisher, the editors and the reviewers. Any product that may be evaluated in this article, or claim that may be made by its manufacturer, is not guaranteed or endorsed by the publisher.

## Footnotes

1
http://www.ncbi.nlm.nih.gov/BLAST

## Supplementary Material

Supplementary Figure 1 Biocrust samples from **(A)** “psyllium” treatment and **(B)** “1:100 biocrust + psyllium” treatments, cultured in solid media specific for cyanobacteria (BG11).
