# Supplementary figures and images for: Biocrust Amendments to Topsoils Facilitate Biocrust Restoration in a Post-mining Arid Environment
Source: Front Microbiol. 2022 Jul 26;13:882673. doi: 10.3389/fmicb.2022.882673 (PMC9360975; doi:10.3389/fmicb.2022.882673)

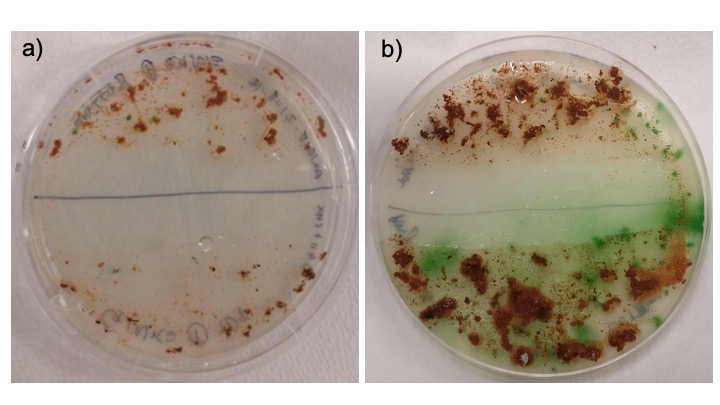

Supplement: Supplementary Figure 1 — Biocrust samples from (A) “psyllium” treatment and (B) “1:100 biocrust + psyllium” treatments, cultured in solid media specific for cyanobacteria (BG11). [file Image_1.JPEG]
